# Editorial for “Positive Computing: A New Partnership Between Psychology, Social Sciences and Technologists”

**DOI:** 10.1186/s13612-016-0047-1

**Published:** 2016-07-04

**Authors:** Rafael A. Calvo, Dianne Vella-Brodrick, Pieter Desmet, Richard M. Ryan

**Affiliations:** University of Sydney, Sydney, Australia; University of Melbourne, Melbourne, Australia; Delft University of Technology, Delft, Netherlands; Australian Catholic University, Sydney, Australia; Rochester University, Rochester, USA

In 2016, almost three and a half billion people, or 46 % of the world population, have an internet connection (internetlivestats 2016). The number of mobile phone users is even higher: more than four and a half billion people own a mobile (or smart) phone (statista.com 2016). Interactive technology is ubiquitous, influencing the daily practices of many people all over the planet. Technology especially permeates the lives of young people whereby those aged 8–18 years spend more time on electronic screens than they do with their parents or at school (Rideout et al. 2010).

Although interactive technologies afford conveniences and efficiencies, the overall contribution of this technology to wellbeing has been a topic of ongoing debate. Some highlight how new technologies inform, liberate and enrich our lives, whereas others suggest that the new technologies too often impoverish our experiences and self-regulation of behaviours, distracting us from relationships and compromising health-promoting activities such as physical activity and sleep. These concerns come at a time when researchers are identifying increases in mental illnesses like depression and anxiety particularly for young people (Sawyer et al. 2012). Some people are asking: *now that we have all this new technology why aren’t we happier?*

The fact is that much of today’s technology was not designed with an explicit focus on wellbeing, nor was it informed by knowledge about the conditions that facilitate it. In fact, we often do not understand the impact of new technologies on wellness until well after they come to market, as we see how people use or abuse new devices and applications. Yet with careful forethought and design, there is the possibility of using designing technology to foster well-being. Well-designed programs and devices can augment people’s physical and metal wellness and, given the sheer numbers of people who use interactive technologies, do so at a scale that traditional “on the ground” interventions cannot capture.

Yet if technology is to be recruited in an effort to improve worldwide wellbeing, then we need new partnerships between psychologists, social scientists, designers and engineers. A broadening in the interdisciplinarity of technology design will enable us to both better understand the psychological and behavioural impact of new technologies and applications, and to develop basic expectations for their design and implementation.

One way forward is to use indicators of wellbeing identified in *positive psychology* research to guide and inform design (Seligman 2011). Leading scholars in positive psychology have, for example, developed metrics for positive emotions, engagement, relationships, meaning and accomplishment reflective of critical components of well-being. Thus the impact of technology on such positive outcomes can be systematically studied with respect to their effects on such indicators of wellness. In addition, some approaches, such as self-determination theory (SDT; Ryan and Deci 2000) look at how technologies impact people’s satisfaction or frustration of basic psychological needs such as autonomy, competence and relatedness, and thus affect both motivation and wellness (Rigby and Ryan 2011). Key goals are to leverage technology to enable greater experiences of choice, connectivity and opportunities for development and mastery.

This new area has been called *Positive Computing* (Calvo and Peters 2014) and it is the focus of this special issue. Positive Computing is an emergent and growing field within human–computer interaction. It shares core goals with work in Positive Design (Desmet and Pohlmeyer 2013) in the industrial design space, and in Positive Technologies (Botella et al. 2012). It also shares the goals of Positive Psychology (Huppert and So 2013) in attempting to understand the factors in everyday life that can enable and enhance human flourishing.

Although Positive Computing is a new initiative, it grows from a deeper history. The decades between 1950 and 1990 saw the creation and blossoming of partnerships between many disciplines including linguistics, neuroscience, psychology, philosophy and what was then the newly-formed field of “cognitive sciences”. Although advances in artificial intelligence are possibly the best-known outcome of this multidisciplinary collaboration, its impact was transformational in other ways. Particularly relevant here is the emergence of *Human Factors*, or ergonomics, the discipline that aims to understand the interaction among humans and technology, in which the collaboration between psychology and designers lead to the development of a set of new practices that make technologies safer, more efficient and easier to use, optimizing overall performance and productivity (APA 2016). The “factors” in this discipline refer to physical, cognitive or behavioural properties of individuals and groups that may influence the quality of a technology. Think, for example, about the properties of your body that need to be taken into account to design a comfortable chair, or the cognitive abilities that limit how much information you can retain in the short term. Research at this interface between behavioural science, psychology and technology has led to crucial advances in the design of equipment, systems and working methods that improve comfort, health, safety, reliability, and productivity. It stands as an example of how design can be focused on enhancing both wellness and functionality.

Given the high level of concern, particularly in developed countries, about the negative impact that many productivity-driven technological changes may be having on psychological wellbeing, a new emphasis on the technology-wellness relationship is warranted. Yet increasing the psychological wellbeing of populations is a complex and ill-defined problem and a topic for scholarly investigation for millennia. Today, most scholars agree that the clinical model of wellbeing, which defines it simply as the absence of illness, is insufficient (Keyes 2007). To fill the gap, researchers in areas like positive psychology study factors that characterize people who are thriving, and how these can be fostered via psychological interventions to improve wellbeing in those who are not (Vella-Brodrick 2012). We can make an analogy with Human Factors, which has generated advanced theory, principles, and methods that support designers in optimizing human-technology interactions by minimizing unwanted negative health and wellbeing effects. But minimizing negative wellbeing does not equal maximizing positive wellbeing.

Over the past decade, designers had already begun to look beyond basic ergonomics and usability and the notion of designing for a positive “user experience” took hold. However, even with an industry now very well-versed in designing for factors like user satisfaction and pleasure, productivity still dominates many of our measures of success. This is probably not only because it’s still easy to measure aspects like “time on task”, but also because as consumers we still attribute great value to increased comfort and productivity. Even self-help and technologies intended to improve wellness often do so based on productivity paradigms, as they encourage us to increase or optimize behaviours like exercise and sleep.

The advancement of future technologies calls for an intelligent and sensitive integration of wellbeing factors into the design of everyday software and products. Although, to date, most examples of design for wellbeing exist as dedicated psychological interventions, these technologies allow us to better understand what is possible (Gaggioli et al. 2016). The reach of dedicated tools may be limited to those actively seeking help, but they are critical in establishing a foundation of research in effective design strategies and psychological impact. The 21 authors who contributed to this issue explore, demonstrate, and discuss the multidisciplinary approaches in Positive Computing. In the diversity of topics and approaches, the resulting eight papers demonstrate the breath of opportunities released when embracing active collaborations between psychologists, computer scientists, and designers.

The paper by Evangelos Karapanos, Ruben Gouveia, Marc Hassenzahl and Jodi Forlizzi, “*Wellbeing in the making: Peoples’ experiences with wearable activity trackers*”, explores how wearable activity trackers create and mediate meaningful experiences in everyday life. While most commercially available trackers employ competition as the primary mode of social exchange, their study showed that tracking involves much more nuanced socially motivated experiences, including a sense of belonging, social support, and bonding. Their findings demonstrate that in a wellbeing-oriented perspective, a mere focus on effectiveness does not suffice. Instead, it requires a deeper understanding of how technology can mediate meaningful experiences in everyday life. Consequently, the ‘tone of voice’ of technologies should be designed to meet the needs of the user.

In “*Communication styles of interactive tools for self*-*improvement”,* Jasmin Niess and Sarah Diefenbach demonstrate that the relevance of this tone of voice, or the communication style, should not be underestimated. Often, just the way products ‘talk to us’ has an important impact on our psychological response and our motivation to use them. Their study shows that communication styles such as helpful-cooperative, rational-distanced, and critical-aggressive have varying emotional impact on people using self-improvement tools, and influence the degree of successful change.

In the dialog between technology and users, not only style but also timing is a crucial factor in effective communication. In their paper “*Kind and grateful”: A context*-*sensitive smartphone app utilizing inspirational content to promote gratitude*”, Asma Ghandeharioun, Asaph Azaria, Sara Taylor and Rosalind Picard, report a study in which they evaluated an active intervention app that cultivates gratitude feelings with the use of inspiring content, such as positive images and famous quotes. Unique in their approach is that the app uses smartphone sensors to find the optimal time for triggering key moments in the intervention and motivational messages, based on location changes, social proximity, and activity levels. The results indicate that timing does matter: The more successful times for eliciting expressions of gratitude tend to be shortly after a social experience and shortly after settling into a new location. We have as much to learn from positive psychological interventions that incorporate the physical world as we do from digital versions.

In “*Form matters: Design creativity in positive psychological interventions*” Pieter Desmet and Maria Sääksjärvi explore how non-digital objects (colorful key ring coins) can be designed as a form of positive psychology intervention. The study compared the wellbeing impact of three conditions in which people were asked to engage in happiness enhancing activities by either using a set of key ring coins, using a paper version, or as part of a control group. Their study shows that while the majority of available behavioral intervention technology relies on screens (computers, tablets, phones), tangible products represent an untapped potential for bringing positive psychology to the everyday lives of many people.

“*Promoting Positive Affect through Smartphone Photography*” by Yu Chen, Gloria Mark and Sanna Ali, explores the wellbeing impact of taking daily pictures with a smartphone. Their four-week study showed that any daily photo-taking with the intent to increase one’s happiness can increase positive affect. Interestingly, they found a theme effect: Taking pictures of things that make the individual happy stimulates one to be more reflective and appreciative of the little pleasures in life, and taking pictures of things that make someone else happier, reduces anxiety and increases a sense of intimacy and belonging. As we record more of our lives, the information produced can be used as part of tools for technology-mediated reflection (TMR), an area of research that may one day inform how platforms like Facebook, can be designed in ways that support our wellbeing. For example, would users benefit most by reflecting on enjoyable experiences when they are in a negative mood?

In “*Technology and reflection: Mood and memory mechanisms for well*-*being*”, Artie Konrad, Simon Tucker, John Crane and Steve Whittaker discuss these relationships between mood, reflection and wellbeing when using technology. With MoodAdaptor, an online system that prompts participants to rate their mood and to write about their memories, they studied how mood and reflection interact in TMR by comparing congruent with incongruent reflection strategies. Their findings reveal a competition between positivity and negativity in our moods and memories, yielding adaptive mechanisms when positivity prevails, and contamination when negativity overshadows.

In “*Design for pride in the workplace*” Yichen Lu and Virpi Roto demonstrate the sheer variety of approaches that can be used to design for wellbeing. Their study, which focused on the experience of pride, investigated 20 examples of experience design by students in a university course. The analysis identified 26 different design strategies, giving a sense of the multiplicity of approaches in which pride can proactively be introduced to the workplace. These strategies fall into four categories defined by two dimensions: self- versus other-focused, and long- versus short-term pride. This study reveals the design strategies for dynamics of pride in the workplace varying from evoking self-achievement in individual interactions with tools to maintaining long-term motivation of self-competence development, and from highlighting one’s contribution in face-to-face collaborative work facilitated by interactive tools to fostering co-experience of organizational pride throughout social events.

Traditionally, design research and practices bypass the awareness of psychology researchers, and psychologists tend to see themselves and their cohorts as the users, rather than the makers of technology, without the power to influence the way technologies are created. Moreover, most engineers have traditionally avoided getting involved in the amorphous hard-to-quantify world of human emotion. A new partnership between psychologists, engineers and designers has the power to overthrow these traditional boundaries, making way for a new era of possibility. For this partnership to be successful, a shared language and even new research methods may be required. The ultimate aim of this special issue is to contribute to the creation of this shared language and to translate multidisciplinary knowledge into practical and effective methods for enhancing well-being for individuals, groups and communities. We have aimed to contribute to this new partnership by bringing together interdisciplinary perspectives and experiences from psychologists, social scientists, designers and software engineers working together towards improving wellbeing. We hope this special issue will help raise awareness of the possibilities and the challenges involved in such an endeavour, and move us closer to a future in which all technology fosters human flourishing.
